# The efficacy of polymyxin B in treating stroke-associated pneumonia with carbapenem-resistant Gram-negative bacteria infections: a multicenter real-world study using propensity score matching

**DOI:** 10.3389/fphar.2025.1413563

**Published:** 2025-03-20

**Authors:** Hai-Hui Zhuang, Qi-Hua Chen, Wei Wang, Qiang Qu, Wei-Xin Xu, Qin Hu, Xiao-Li Wu, Ying Chen, Qing Wan, Tian-Tian Xu, Wen-Ming Long, Yue Luo, Hai-Nan Zhang, Jian Qu

**Affiliations:** ^1^ Department of Pharmacy, The Second Xiangya Hospital, Institute of Clinical Pharmacy, Central South University, Changsha, China; ^2^ Department of Neurology, The Second Xiangya Hospital, Central South University, Changsha, China; ^3^ Department of Pharmacy, Xiangya Hospital, Central South University, Changsha, China; ^4^ Department of Pharmacy, The Second Affiliated Hospital of Guangzhou Medical University, Guangzhou, China; ^5^ Department of Pharmacy, Renmin Hospital, Wuhan University, Wuhan, China; ^6^ Department of Pharmacy, The First Affiliated Hospital of Nanchang University, Nanchang, China; ^7^ Department of Pharmacy, The Second People’s Hospital of Huaihua, Huaihua, China; ^8^ Department of Pharmacy, The People’s Hospital of Liuyang, Liuyang, China; ^9^ Hunan Key Laboratory of the Research and Development of Novel Pharmaceutical Preparations, Changsha Medical University, Changsha, China

**Keywords:** stroke-associated pneumonia, polymyxin B, carbapenem-resistant gram-negative bacteria, clinical efficacy, microbial efficacy

## Abstract

**Objectives:**

Infection with Carbapenem-resistant Gram-negative bacteria (CR-GNB) poses further challenges in treating stroke-associated pneumonia (SAP) patients. This multicenter retrospective study aimed to evaluate the efficacy of polymyxin B (PMB) in CR-GNB-infected SAP patients and to identify factors that may influence its effectiveness.

**Methods:**

From 1 September 2019, and 30 December 2022, a total of 196 CR-GNB-infected SAP patients from five hospitals in China were included in the study based on specific criteria. Demographics and clinical data were obtained from the electronic medical records. Propensity score matching (PSM) was used to minimize the effect of potential confounding variables. Univariate analysis and multivariate logistic analysis were performed to identify risk factors affecting microbial efficacy.

**Results:**

Among the 196 SAP patients infected with CR-GNB, 24.5% received PMB combined inhalation and 75.5% received non-combined inhalation treatment. The clinical success rate was 68.9%, with 25.5% achieving microbial efficacy within 7 days and 37.8% achieving microbial cure. The 30-day all-cause mortality rate was 14.8%. The incidence of acute kidney injury was 34.7%. After adjustment by propensity score matching, the PMB combined inhalation group exhibited significantly higher microbial efficacy compared to the non-combined inhalation group (46.7% vs. 26.7%, p = 0.049). Multivariate logistic analysis identified multi-site infections and Carbapenem-resistant *Pseudomonas aeruginosa* infection as independent risk factors for microbial efficacy.

**Conclusion:**

Combined inhalation of PMB demonstrated superior effectiveness in microbial clearance compared to non-combined inhalation in treating CR-GNB-infected SAP patients. We recommend aerosol combined inhalation of PMB and suggest developing personalized PMB-based regimens for individual patients to enhance treatment outcomes.

## 1 Introduction

Stroke-associated pneumonia (SAP) is a common complication that occurs within the first week after a stroke, affecting 7%–38% of stroke patients (Badve et al., 2019). Recent studies have confirmed that SAP is associated with higher mortality, worse outcomes in survivors, and longer hospital stays (Ali et al., 2018; Zhao et al., 2021). Patients with acute stroke are particularly susceptible to disruptions in their oral microbiota due to reduced chewing, salivation, swallowing, and oral hygiene (Grossmann et al., 2021; Zhao et al., 2021). The presence of oral pathogens in stroke patients is associated with a poor prognosis and can lead to aspiration pneumonia (Grossmann et al., 2021; Zhao et al., 2021). SAP often involves a mix of pathogens associated with early-onset nosocomial pneumonia and community-acquired aspiration syndrome (Westendorp et al., 2022). A systematic review of 15 studies identified the commonly detected pathogens as Enterobacteriaceae (*Klebsiella pneumoniae* and *Escherichia coli*), *Staphylococcus aureus, Pseudomonas aeruginosa*, *Acinetobacter baumannii*, and *Streptococcus pneumoniae* (Kishore et al., 2018). These pathogens are similar to those commonly found in hospital-acquired pneumonia (HAP) or community-acquired pneumonia (CAP) (Zhao et al., 2021). Mechanical ventilation (MV) is used in critically ill patients to maintain normal gas exchange. However, the mechanical force exerted by MV can damage the normal airway barrier and impair the lower respiratory tract’s ability to eliminate microbes (Mandell and Niederman, 2019; Zhao et al., 2021). Therefore, MV is a risk factor for SAP, and critically ill patients on MV are at a high risk of developing SAP (Mandell and Niederman, 2019).

Infections caused by carbapenem-resistant Gram-negative bacteria (CR-GNB) have drastically increased over the past decade and have become a major global public health challenge with high mortality rates (Lu et al., 2021a). Treatment options for CR-GNB infections are limited, especially in China, where polymyxins, tigecycline, and ceftazidime-avibactam are available (Qu et al., 2022). Polymyxins are a group of chemicals derived from *Paenibacillus polymyxa* and were clinically useful in the 1950s (Nation et al., 2014). Currently, three different forms of polymyxins are available in the international market: colistimethate sodium (CMS), polymyxin B sulfate (PMB), and colistin sulfate, with the latter being unique to the Chinese market (Lu et al., 2021b). Polymyxin B is a polypeptide antibiotic with strong activity against most CR-GNB infections (Lu et al., 2021b; Qu et al., 2022). However, its clinical use has been limited due to side effects, especially nephrotoxicity (Wu et al., 2022). Recent studies have found that PMB can effectively treat extensively drug-resistant Gram-negative ventilator-associated pneumonia, particularly when inhaled (Liu et al., 2022; Ye et al., 2022). However, there are few studies on the use of PMB in the treatment of CR-GNB-infected SAP. In this multicenter retrospective study, we aim to investigate the efficacy of PMB in SAP infections.

## 2 Patients and methods

### 2.1 Ethics

The study received approval from the Ethics Committees of the Second Xiangya Hospital of Central South University (LYF-2020021) and was conducted in accordance with the ethical standards of the Helsinki Declaration (1964). Due to the retrospective nature of the study, written informed consent was waived by the ethics committee.

### 2.2 Patients

The study included patients who were admitted to several hospitals, namely, the Second Xiangya Hospital of Central South University (a 3,500-bed general hospital), Xiangya Hospital of Central South University (a 3,500-bed general hospital), the First Affiliated Hospital of Nanchang University, the Second Affiliated Hospital of Guangzhou Medical University (a 2,500-bed general hospital), and Renmin Hospital of Wuhan University (a 3,500-bed general hospital) between 1 September 2019, and 30 December 2022. Patients were eligible for inclusion if they met the following criteria: (a) had SAP (diagnosed within 7 days after stroke); (b) were diagnosed with CR-GNB pneumonia based on two consecutive bronchial secretions or bronchoalveolar lavage cultures, antimicrobial susceptibility testing, clinical symptoms, and lung imaging data; (c) received intravenous PMB for ≥72 h; and (d) had available data to evaluate the effectiveness of PMB. Patients were excluded if they were pregnant, under 18 years of age, the antibiotic susceptibility test indicated cases where CR-GNB strains were resistant to PMB.

Demographics and clinical characteristics, such as age, gender, baseline comorbidities, infection sites, Acute Physiology and Chronic Health Evaluation II (APACHE II) score, details of antibiotic use, and inflammatory indicators, were retrospectively obtained from electronic records. All collected data were anonymized.

### 2.3 Outcomes and definitions

Patients were diagnosed with SAP if they developed lower respiratory tract infections within the first 7 days after the index stroke, based on the modified Centers for Disease Control and Prevention criteria (Smith et al., 2015). The diagnosis of SAP was retrospectively determined by two neurologists (Qi-Hua Chen and Wei Wang). Clinical outcomes were assessed by two physicians and categorized as clinical success or clinical failure. Clinical cure was defined as symptom resolution or significant improvement following PMB therapy, including a composite of survival, hemodynamic stability, body temperature <38°C, improved inflammatory indicators, and a stable or improved PaO_2_/FiO_2_ ratio (King et al., 2017). Microbial cure was defined as the absence of the initially isolated pathogen from the site of infection and the disappearance of clinical pneumonia manifestation. In cases of multi-site CR-GNB infection, microbial cure was defined as the absence of detection at all infection sites in the patient. Thirty-day all-cause mortality was defined as death from any cause within 30 days of PMB treatment.

Baseline serum creatinine was measured on the day that PMB was initiated. Normal renal function was defined as a glomerular filtration rate (GFR) ≥60 mL/min per 1.73 m^2^. Renal injury was evaluated using the RIFLE (risk, injury, failure, loss, end-stage kidney disease) criteria, which compared the highest serum creatinine value observed during PMB therapy with the baseline creatinine level (Bellomo et al., 2004). Acute kidney injury (AKI) was defined according to the Kidney Disease: Improving Global Outcomes (KDIGO) criteria, which require either a 0.3 mg/dL increase in serum creatinine within 48 h or a 50% increase in serum creatinine within 7 days. Creatinine clearance (CrCL) was calculated using the Cockcroft–Gault equation.

### 2.4 Microbiology

Bacterial species were identified using a matrix-assisted laser desorption ionization-time of flight mass spectrometer (bioMerieux, Marcyl ‘Etoile, France). Antimicrobial susceptibilities and the minimum inhibitory concentration (MIC) breakpoints for most antibiotics were determined by the broth microdilution method using the VITEK^®^2 system (bioMérieux, Marcy-l’Étoile, France PMB) and were interpreted based on the Clinical and Laboratory Standard Institute (CLSI) 2020 guideline (Institute, 2020). The MIC breakpoint for tigecycline and PMB was interpreted by European Committee on Antimicrobial Susceptibility Testing 2020 criteria (Satlin et al., 2020). Carbapenem resistance was defined as a MIC ≥4 mg/L of imipenem or meropenem (Codjoe and Donkor, 2017).

### 2.5 Statistical analysis

Statistical analysis was performed using SPSS version 21.0 (IBM, Armonk, NY, United States). Quantitative data were presented as mean ± standard deviations (SD) or medians (interquartile range, IQR). For comparisons between the two groups, Student's t-tests or Mann–Whitney U tests were used. Categorical data were presented as numbers of cases and percentages and were analyzed by χ^2^ tests or Fisher’s exact tests. A group was established that received intravenous and nebulized administration of PMB, and a group that only received intravenous administration of PMB. Univariate analysis was used to compare the differences between the two groups. In order to minimize the impact of potential confounding factors, we used SPSS 21.0 software to perform 1:1 propensity score matching (PSM) for variables with p < 0.1 between the two groups. The matching tolerance was set at 0.2, and the order of cases was randomly arranged when drawing matched items. Multivariate logistic regression analysis was performed to identify potential independent predictors of PMB efficacy predictors. Factors with p-values <0.1 in univariate analysis were included in the multivariate logistic regression analysis. Significance was defined as p-values <0.05 (two-tailed).

## 3 Results

### 3.1 Baseline clinical characteristics

**FIGURE 1:**
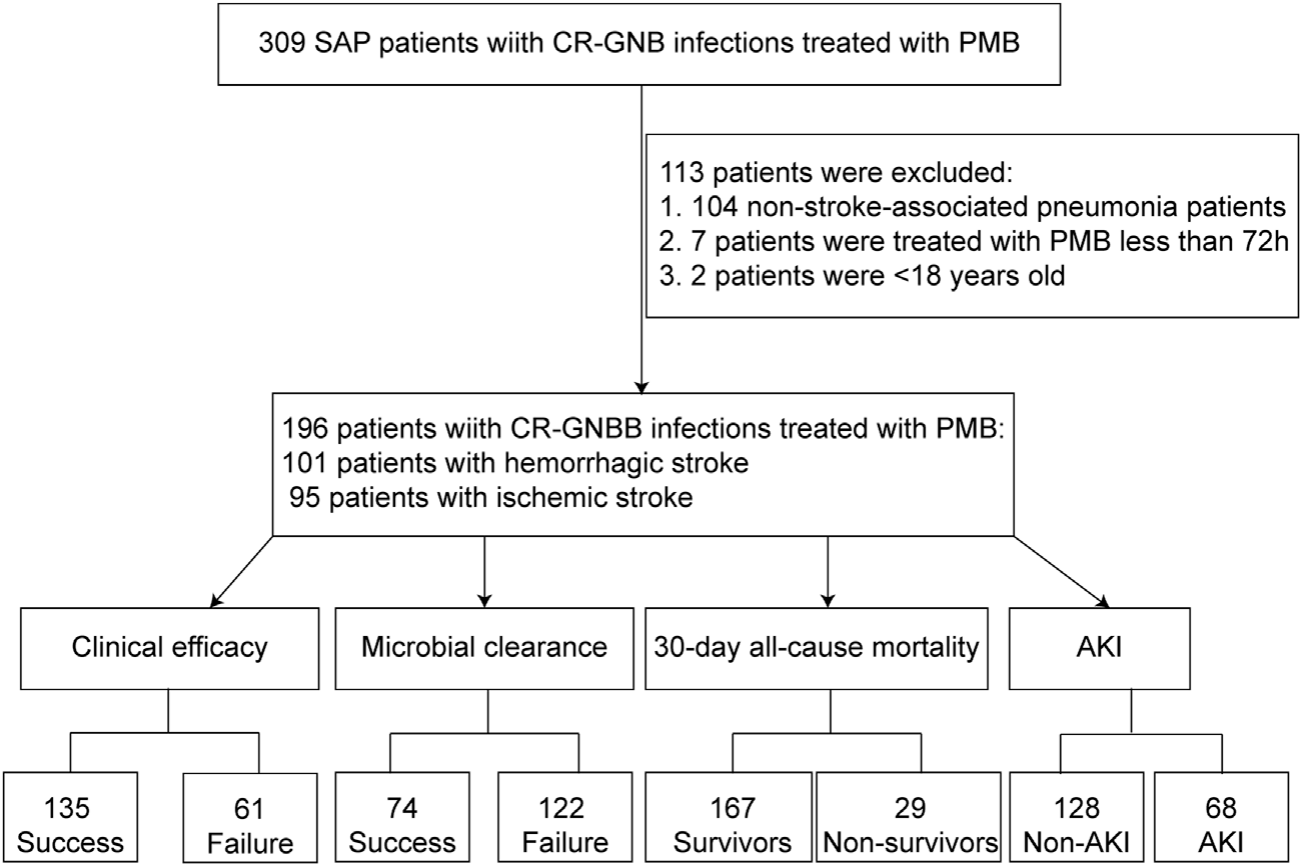
The flowchart of the patients included in the study.

According to the inclusion and exclusion criteria, a total of 196 patients who were treated with intravenous PMB and met the criteria were included in our study. (Figure 1). A total of 66 patients were from the Second Xiangya Hospital of Central South University, 60 patients were from Xiangya Hospital of Central South University, 30 patients were from the First Affiliated Hospital of Nanchang University, 21 patients were from the Second Affiliated Hospital of Guangzhou Medical University, 15 patients were from Renmin Hospital of Wuhan University, 3 patients were from Liuyang People’s Hospital, and 1 patient was from the Second People’s Hospital of Huaihua City. The demographic and clinical characteristics are summarized in Table 1. The medium age was 64.0 (IQR 51.0–75.8) years, with the majority patients being male gender (79.1%). Among the 196 patients, 81.6% were admitted to ICU, 71.4% received mechanical ventilation, and 44.9% received vasoactive drugs for treatment. The duration of hospitalization was 34.5 (IQR 20.3–55.0) days, and the APACHE II score was 19.6 ± 5.9. Before PMB treatment, patients’ creatinine was 72.0 (IQR 52.1–112.6). The sepsis/septic shock rate was 27.0%. Patients with ischemic stroke accounted for 48.5% and hemorrhagic stroke accounted for 51.5%. Among the infected sites, 25.0% were multi-site infections, 9.7% were central nervous system infections, 9.2% were blood infections, 8.7% were urinary tract infections, 2.0% were abdominal infections, and 2.0% were skin and soft tissue infections.

**TABLE 1:** Clinical characteristics and efficacy of PMB inhalation in the treatment of stroke-associated pneumonia caused by CR-GNB infection (before PSM).

Demographics and clinical characteristics	Total (N = 196)	Non- combined inhalation (N = 148)	Combined inhalation (N = 48)	P-value
Age (years)	64.0 (51.0–75.8)	61.0 (50.0–74.5)	71.0 (51.3–78.0)	**0.049**
Gender (male)	155 (79.1%)	118 (79.7%)	37 (77.1%)	0.695
Mechanical ventilation	140 (71.4%)	97 (65.5%)	43 (89.6%)	**0.001**
Vasoactive drugs	88 (44.9%)	59 (39.9%)	29 (60.4%)	**0.013**
ICU administration	160 (81.6%)	115 (77.7%)	45 (93.8%)	**0.013**
Hospital stays (days)	34.5 (20.3–55.0)	19.4 ± 5.7	20.2 ± 6.4	0.404
APACHE II score	19.6 ± 5.9	19.4 ± 5.7	20.2 ± 6.4	0.404
Creatinine before treatment (µmol/L)	72.0 (52.1–112.6)	70.6 (50.4–102.8)	81.5 (55.5–140.4)	0.116
Sepsis/septic shock	53 (27.0%)	31 (20.9%)	22 (45.8%)	**0.001**
Comorbidity				
Stroke type Ischemic	95 (48.5%)	66 (44.6%)	29 (60.4%)	**0.057**
Hemorrhagic	101 (51.5%)	82 (55.4%)	19 (39.6%)	
Hypoproteinemia	56 (28.6%)	39 (26.4%)	17 (35.4%)	0.227
Urinary system disease	29 (14.8%)	20 (13.5%)	9 (18.8%)	0.375
Diabetes mellitus	50 (25.5%)	36 (24.3%)	14 (29.2%)	0.504
Digestive system diseases	62 (31.6%)	45 (30.4%)	17 (35.4%)	0.516
Cardiovascular diseases	133 (67.9%)	98 (66.2%)	35 (72.9%)	0.388
Malignancy	25 (12.8%)	19 (12.8%)	6 (12.5%)	0.951
Infection sits				
Multi-site infections	49 (25.0%)	35 (23.6%)	14 (29.2%)	0.443
Blood	18 (9.2%)	11 (74%)	7 (14.6%)	0.136
Abdominal	4 (2.0%)	2 (1.4%)	2 (4.2%)	0.252
Urinary tract	17 (8.7%)	11 (7.4%)	6 (12.5%)	0.278
Central nervous system	19 (9.7%)	16 (10.8%)	3 (6.3%)	0.517
Sin and soft tissue	4 (2.0%)	4 (2.7%)	0 (0.0%)	0.574
Pathogens				
CRKP	72 (36.7%)	57 (38.5%)	15 (31.3%)	0.364
CRPA	54 (27.6%)	44 (29.7%)	10 (20.8%)	0.231
CRAB	142 (72.4%)	105 (70.9%)	37 (77.1%)	0.408
Other CREs	8 (4.1%)	5 (3.4%)	3 (6.3%)	0.650
Multiple CR-GNBs	70 (35.7%)	56 (37.8%)	14 (29.2%)	0.276
PMB Treatment				
Loading dose (%)	130 (66.3%)	91 (61.5%)	39 (81.3%)	**0.012**
Loading dose (mg)	100 (100.0–125.0)	100.0 (100.0–100.0)	100.0 (100.0–150.0)	**0.062**
Maintenance dose (mg)	75.0 (50.0–75.0)	50.0 (50.0–75.0)	75.0 (50.0–75.0)	0.475
Loading dose≥2 mg/kg	71 (36.2%)	49 (33.1%)	22 (45.8%)	0.111
Maintenance dose≥1.25 mg/kg	87 (44.4%)	63 (42.6%)	24 (50.0%)	0.368
Treatment course (days)	10.3 (6.5–15.0)	10.0 (6.0–14.0)	11.0 (7.3–18.0)	0.205
Cumulative dose (mg)	1,325.0 (813.8–2,168.8)	1,300.0 (806.3–1850.0)	1,438.5 (820.0–2,465.0)	0.234
Number of antibiotics	1.3 ± 0.7	1.2 ± 0.6	1.5 ± 0.8	**0.015**
β-lactam	82 (41.8%)	64 (43.2%)	18 (37.5%)	0.483
Tigecycline	43 (21.9%)	27 (18.2%)	16 (33.3%)	**0.028**
Carbapenem	92 (46.9%)	69 (46.6%)	23 (47.9%)	0.876
Only PMB	37 (18.9%)	34 (23.0%)	3 (6.3%)	**0.018**
Treatment outcome				
Clinical efficacy	135 (68.9%)	99 (66.9%)	36 (75.0%)	0.292
7-day microbial efficacy	50 (25.5%)	37 (25.0%)	13 (27.1%)	0.774
Microbial cure	74 (37.8%)	51 (34.5%)	23 (47.9%)	0.095
30-day all-cause mortality	29 (14.8%)	21 (14.2%)	8 (16.7%)	0.674
Bacterial clearance time	8.0 (6.0–15.0)	8.0 (5.0–14.0)	8.0 (6.0–17.5)	0.485
Survival period (days)	27.0 ± 7.4	27.3 ± 7.0	26.3 ± 8.6	0.473
AKI rate	68 (34.7%)	46 (31.1%)	22 (45.8%)	0.062
Inhalation therapy regimen (days)	0.0 (0.0–0.0)	0.0 (0.0–0.0)	8.0 (5.0–13.8)	-

PMB, polymyxin B; AKI, acute kidney injury; ICU, intensive care unit; CR-GNB, carbapenem-resistant Gram-negative bacteria; CRKP, carbapenem-resistant *Klebsiella pneumoniae*; CRAB, Carbapenem-resistant *acinetobacter baumannii*; CRPA, Carbapenem-resistant *pseudomonas aeruginosa*.

Bold annotations indicate statistical differences.

A total of 35.7% of patients (N = 70) had multiple CR-GNB infections. The most common pathogenic bacteria were Carbapenem-resistant *Acinetobacter baumannii* (CRAB) (N = 142; 72.4%), followed by Carbapenem-resistant *K. pneumoniae* (CRKP) (N = 72; 36.7%), Carbapenem-resistant *Pseudomonas aeruginosa* (CRPA) (N = 54; 27.6%), and other Carbapenem-resistant *Enterobacteriaceae* (CREs) (N = 8; 4.1%). The sensitivity rate of PMB was 98.9%, and only three strains, including one CRAB, one CRPA, and one CRKP strain, were resistant to PMB. For most CR-GNB stains, the MIC values of PMB were 1 mg/L or ≤0.5 mg/L. A total of 169 isolates of CRKP and CRAB underwent susceptibility testing for tigecycline. The MIC values of tigecycline for 113 isolates were ≤1 mg/L. For 31 isolates, the MIC values were 2 mg/L, while 25 isolates had MIC values greater than 8 mg/L.

### 3.2 Medications and outcomes

Out of the 196 patients included, 24.5% of patients (N = 48) were treated with inhalation of PMB, the specific method of inhalation administration is illustrated in Supplementary Figure S1. Only 66.3% of patients (N = 130) received a PMB loading dose with a medium 100 (IQR 100.0–125.0) mg. The maintenance dose was 75.0 (IQR 50.0–75.0) mg q12 h. The loading dose>2 mg/kg accounted for 36.2% of patients (N = 71). The maintenance dose>1.25 mg/kg accounted for 44.4% (N = 87). The treatment course was 10.3 (IQR 6.5–15.0) days. PMB monotherapy was used in 18.9% of patients, and the combination therapy included carbapenem (46.9%), β-lactam drugs (41.8%), and tigecycline (21.9%) (Table 1).

For CR-GNB-infected SAP patients, the clinical success rate of PMB treatment was 68.9%. The 7-day microbial efficacy and microbial cure were 25.5% and 37.8%, respectively. The median bacterial clearance time was 8.0 (IQR 6.0–15.0) days. The 30-day all-cause mortality was 14.8%, and the average survival time was 27.0 ± 7.4 days. The incidence of AKI was 34.7% (Table 1), with 42 (21.4%) patients in stage Risk (R), 16 (8.2%) patients in stage Injury (I), and 10 (5.1%) patients in stage Failure (F).

### 3.3 Clinical characteristics of patients in the propensity score matching cohort

We divided 196 SAP patients who received intravenous PMB into a combined inhalation group and a non-combined inhalation group, with 48 patients (24.5%) in the combined inhalation group. PMB nebulized administration is carried out via inhalation using ultrasonic nebulization. There were significant differences between the two groups on some baseline clinical characteristics, including age, mechanical ventilation, vasoactive drugs, ICU administration, and sepsis/septic shock. Moreover, the proportion of PMB loading dose, combination of tigecycline, and number of antibiotics were higher in the PMB combined inhalation group than in the non-combined inhalation group (p < 0.05). PMB monotherapy was more frequently used in the non-combined inhalation group than in the combined inhalation group (p = 0.018) (Table 1).

There were imbalances in the baseline variables between the two groups in the original cohort. The propensity score matching was applied to identify a cohort with similar baseline characteristics in the two groups. After PSM, 45 patients receiving the PMB combined inhalation therapy were matched with 45 patients receiving PMB non-combined inhalation therapy. After PSM adjustment, the risk factors of the two groups were more balanced (p > 0.05 for most factors) (Table 2).

**TABLE 2:** Clinical characteristics and efficacy of PMB inhalation in the treatment of stroke-associated pneumonia caused by CR-GNB infection (after PSM).

Demographics and clinical characteristics	Total (N = 90)	Non-combined inhalation (N = 45)	Combined inhalation (N = 45)	P-value
Age (years)	68.0 (54.8–77.0)	68.0 (55.5–76.0)	69.0 (53.0–77.5)	0.862
Gender (male)	71 (78.9%)	37 (82.2%)	34 (75.6%)	0.438
Mechanical ventilation	79 (87.8%)	39 (86.7%)	40 (88.9%)	0.748
Vasoactive drugs	46 (51.1%)	20 (44.4%)	26 (57.8%)	0.206
ICU administration	84 (93.3%)	42 (93.3%)	42 (93.3%)	1.000
Hospital stays (days)	36.5 (19.0–55.5)	39.0 (24.0–56.0)	29.0 (19.0–56.0)	0.319
APACHE II score	19.4 (19.4–21.0)	19.4 (18.7–127.0)	19.4 (19.4–23.0)	0.688
Creatinine before treatment (µmol/L)	81.5 (54.9–129.7)	83.0 (54.9–127.0)	80.3 (55.0–133.8)	0.796
Sepsis/septic shock	35 (38.9%)	16 (35.6%)	19 (42.2%)	0.517
Comorbidity				
Stroke type Ischemic	53 (58.9%)	27 (60.0%)	26 (57.8%)	
Hemorrhagic	37 (41.1%)	18 (40.0%)	19 (42.2%)	0.830
Hypoproteinemia	29 (32.2%)	13 (28.9%))	16 (35.6%)	0.499
Urinary system disease	16 (17.8%)	7 (15.6%)	9 (20.0%)	0.581
Diabetes mellitus	24 (26.7%)	11 (24.4%)	13 (28.9%)	0.634
Digestive system diseases	32 (35.6%)	16 (35.6%)	16 (35.6%)	1.000
Cardiovascular diseases	60 (66.7%)	28 (62.2%)	32 (71.1%)	0.371
Malignancy	9 (10.0%)	4 (8.9%)	5 (11.1%)	1.000
Infection sits				
Multi-site infections	23 (25.6%)	10 (22.2%)	13 (28.9%)	0.468
Blood	11 (12.2%)	5 (11.1%)	6 (13.3%)	0.748
Abdominal	2 (2.2%)	1 (2.2%)	1 (2.2%)	1.000
Urinary tract	10 (11.1%)	4 (8.9%)	6 (13.3%)	0.737
Central nervous system	6 (6.7%)	3 (6.7%)	3 (6.7%)	1.000
Sin and soft tissue	0 (0.0%)	0 (0.0%)	0 (0.0%)	-
Pathogens				
CRKP	31 (34.4%)	18 (40.0%)	13 (28.9%)	0.267
CRPA	24 (26.7%)	16 (35.6%)	8 (17.8%)	0.057
CRAB	66 (73.3%)	31 (68.9%)	35 (77.8%)	0.340
Other CREs	6 (6.7%)	3 (6.7%)	3 (6.7%)	1.000
Multiple CR-GNBs	32 (35.6%)	20 (44.4%)	12 (26.7%)	0.078
PMB Treatment				
Loading dose (%)	74 (82.2%)	37 (82.2%)	37 (82.2%)	1.000
Loading dose (mg)	100.0 (100.0–150.0)	100.0 (100.0–150.0)	100.0 (100.0–150.0)	0.669
Maintenance dose (mg)	75.0 (50.0–75.0)	50.0 (50.0–75.0)	75.0 (50.0–75.0)	0.341
Loading dose≥2 mg/kg	37 (41.1%)	17 (37.8%)	20 (44.4%)	0.520
Maintenance dose≥1.25 mg/kg	42 (46.7%)	20 (44.4%)	22 (48.9%)	0.673
Treatment course (days)	10.0 (6.0–15.0)	10.0 (6.0–14.0)	11.0 (7.0–17.75)	0.196
Cumulative dose (mg)	1,250.0 (801.3–2,200.0)	1,200.0 (712.5–1,650.0)	1,425.0 (830.0–2,437.50)	0.218
Number of antibiotics	1.0 (1.0–2.0)	1.0 (1.0–2.0)	1.0 (1.0–2.0)	0.742
β-lactam	39 (43.3%)	22 (48.9%)	17 (37.8%)	0.288
Tigecycline	31 (34.4%)	17 (37.8%)	14 (31.1%)	0.506
Carbapenem	43 (47.8%)	21 (46.7%)	22 (48.9%)	0.833
Only PMB	4 (4.4%)	1 (2.2%)	3 (6.7%)	0.616
Treatment outcome				
Clinical efficacy	65 (72.2%)	30 (66.7%)	35 (77.8%)	0.239
7-day microbial efficacy	20 (22.2%)	8 (17.8%)	12 (26.7%)	0.310
Microbial cure	33 (36.7%)	12 (26.7%)	21 (46.7%)	**0.049**
30-day all-cause mortality	18 (20.0%)	10 (22.2%)	8 (17.8%)	0.598
Bacterial clearance time (days)	9.0 (6.0–16.0)	11.0 (5.0–16.0)	8.00 (6.0–16.5)	0.630
30-day Survival period (days)	30.0 (30.0–30.0)	30.0 (30.0–30.0)	30.0 (30.0–30.0)	0.742
AKI rate	37 (41.1%)	17 (37.8%)	20 (44.4%)	0.520
Inhalation therapy regimen (days)	0.5 (0.0–8.0)	0.0 (0.0–0.0)	8.0 (4.5–13.5)	-

PSM, Propensity score matching. The rest of the abbreviations are the same as Table 1.

Bold annotations indicate statistical differences.

In the matched cohort, the median age of patients was 68.0 (IQR 54.8–77.0) years, and 78.9% were male. A total of 53 (58.9%) patients were diagnosed with ischemic stroke, and 37 (41.1%) were diagnosed with hemorrhagic stroke. Among the included patients, 73.3% were infected with CRAB, 34.4% were with CRKP, 26.7% were with CRPA, 6.7% were with other CREs, and 35.6% of them were infected with multiple CR-GNBs. Details of patients’ characteristics in the matched cohort were summarized in Table 2. The overall clinical cure, 30-day all-cause mortality, and AKI incidence in the matched cohort were 72.2%, 20.0%, and 41.0%, respectively. It is worth mentioning that the microbial cure rate was significant higher in the PMB combined inhalation group than in the non-combined inhalation group (46.7% vs. 26.7%, p = 0.049) after PSM.

### 3.4 Risk factors associated the microbial cure of PMB

Next, CR-GNB-infected SAP patients who underwent microbial clearance evaluation after treatment were divided into the clearance group and the non-clearance group. Univariate analysis showed that the proportion of CRAB infection was higher in the microbial cure patients than in the microbial failure patients (83.8% vs. 65.6%, p = 0.006). Conversely, the proportion of CRPA infection and multiple CR-GNB infections was higher in the microbial non-clearance patients than in the microbial clearance patients (36.9% vs. 12.2%, p < 0.001; 41.0% vs. 27.0%, p = 0.048). Additionally, microbiological efficacy was associated with treatment course and cumulative dose (p < 0.05) (Table 3). After PSM, univariate analysis showed that the proportion of hemorrhagic stroke (p = 0.016) and CRPA infection (p = 0.009) was higher in the microbial non-clearance group. And PMB inhalation, treatment course, cumulative dose, number of antibiotics were related to microbial clearance (p < 0.05) (Table 3).

**TABLE 3:** Univariate analysis of microbial efficacy of PMB in the treatment of stroke-associated pneumonia with CR-GNB infection.

Demographics and clinical characteristics	Before PSM			After PSM		
	Clearance (N = 74)	Non-clearance (N = 122)	P-value	Clearance (N = 33)	Non-clearance (N = 57)	P-value
Age (years)	63.5 (50.8–74.0)	65.5 (51.0–76.3)	0.544	64.0 (53.0–72.5)	72.0 (55.5–77.5)	0.187
Gender (male)	56 (75.7%)	99 (1.1%)	0.361	25 (75.8%)	46 (80.7%)	0.580
Mechanical ventilation	56 (75.7%)	84 (68.9%)	0.305	29 (87.9%)	50 (87.7%)	1.000
Vasoactive drugs	32 (43.2%)	56 (45.9%)	0.717	14 (42.4%)	32 (56.1%)	0.210
ICU administration	62 (83.8%)	98 (80.3%)	0.545	31 (93.9%)	53 (93.0%)	1.000
Hospital stays (days)	34.5 (19.8–55.0)	35.0 (21.0–56.3)	0.548	35.0 (21.5–56.0)	40.0 (19.0–56.5)	0.808
APACHE II score	19.4 (19.4–20.0)	19.4 (19.4–19.4)	0.958	19.4 (16.5–20.5)	19.4 (19.4–21.5)	0.299
Creatinine before treatment (µmol/L)	77.9 (51.5–129.5)	71.3 (53.2–108.1)	0.856	83.0 (58.0–133.8)	81.3 (54.4–125.6)	0.516
Sepsis/septic shock	16 (21.6%)	37 (30.3%)	0.183	9 (27.3%)	26 (45.6%)	**0.085**
Comorbidity						
Stroke type						
Ischemic	43 (58.1%)	58 (47.5%)		19 (57.6%)	18 (31.6%)	
Hemorrhagic	31 (41.9%)	64 (52.5%)	0.151	14 (42.4%)	39 (68.4%)	**0.016**
Hypoproteinemia	23 (31.1%)	33 (27.0%)	0.545	14 (42.4%)	15 (26.3%)	0.115
Urinary system disease	9 (12.2%)	20 (16.4%)	0.419	5 (15.2%)	11 (19.3%)	0.620
Diabetes mellitus	19 (25.7%)	31 (25.4%)	0.967	11 (33.3%)	13 (22.8%)	0.277
Digestive system diseases	24 (32.4%)	38 (31.1%)	0.851	12 (36.4%)	20 (35.1%)	0.903
Cardiovascular diseases	52 (70.3%)	81 (66.4%)	0.573	24 (72.7%)	36 (63.2%)	0.353
Malignancy	9 (2.2%)	16 (13.1%)	0.846	4 (12.1%)	5 (8.8%)	0.610
Infection sits						
Multi-site infections	13 (17.6%)	36 (29.5%)	**0.061**	7 (21.2%)	16 (28.1%)	0.472
Blood	5 (6.8%)	13 (10.7%)	0.360	4 (12.1%)	7 (12.3%)	1.000
Abdominal	1 (1.4%)	3 (2.5%)	1.000	1 (3.0%)	1 (1.8%)	1.000
Urinary tract	7 (9.5%)	10 (8.2%)	0.761	4 (12.1%)	6 (10.5%)	1.000
Central nervous system	5 (6.8%)	14 (11.5%)	0.279	2 (6.1%)	4 (7.0%)	1.000
Sin and soft tissue	1 (1.4%)	3 (2.5%)	1.000	0	0	-
Pathogens						
CRKP	23 (21.1%)	49 (40.2%)	0.201	9 (27.3%)	22 (38.6%)	0.276
CRPA	9 (12.2%)	45 (36.9%)	**<0.001**	3 (9.1%)	21 (36.8%)	**0.009**
CRAB	62 (83.8%)	80 (65.6%)	**0.006**	29 (87.9%)	37 (64.9%)	**0.033**
Other CREs	2 (2.7%)	6 (4.9%)	0.698	2 (6.1%)	4 (7.0%)	1.000
Multiple CR-GNB infections	20 (27.0%)	50 (41.0%)	**0.048**	9 (27.3%)	23 (40.4%)	0.212
PMB Treatment						
Inhalation (%)	23 (31.1%)	25 (20.5%)	**0.095**	21 (63.6%)	24 (42.1%)	**0.049**
Inhalation therapy regimen (days)	0.0 (0.0–5.0)	0.0 (0.0–0.0)	**0.125**	3.5 (0.0–10.0)	0.0 (0.0–7.0)	**0.104**
Loading dose (%)	49 (66.2%)	81 (66.4%)	0.980	27 (81.8%)	47 (82.5%)	0.939
The loading dose (mg)	100.0 (100.0–125.0)	100.0 (93.8–105.0)	0.356	100.0 (100.0–150.0)	100.0 (100.0–142.5)	0.456
Maintenance dose (mg)	75.0 (50.0–100.0)	50.0 (50.0–75.0)	**0.052**	75.0 (50.0–95.0)	50.0 (50.0–75.0)	0.102
The loading dose≥2 mg/kg	28 (37.8%)	43 (35.2%)	0.714	14 (42.4%)	23 (40.4%)	0.847
Maintenance dose ≥ 1.25 mg/kg	37 (50.0%)	50 (41.0%)	0.218	17 (51.5%)	25 (26.6%)	0.483
Treatment course (days)	11.0 (8.0–16.0)	10.0 (6.0–14.0)	**0.035**	14.0 (8.0–16.5)	9.0 (5.3–13.5)	**0.008**
Cumulative dose (mg)	1,600.0 (1,207.5–2,431.3)	1,150.0 (718.8–1737.5)	**0.002**	1,650.0 (1,275.0–3,000.0)	1,050.0 (700.0–1750.0)	**0.001**
Number of antibiotics	1.0 (1.0–2.0)	1.0 (1.0–2.0)	0.441	2.0 (1.0–2.0)	1.0 (1.0–2.0)	**0.031**
β-lactam	30 (40.5%)	52 (42.6%)	0.774	16 (48.5%)	23 (40.4%)	0.453
Tigecycline	19 (25.7%)	24 (19.7%)	0.325	14 (42.4%)	17 (29.8%)	0.225
Carbapenem	37 (50.0%)	55 (45.1%)	0.504	18 (54.5%)	25 (43.9%)	0.328
Only PMB	12 (16.2%)	25 (20.5%)	0.458	0 (0.0%)	4 (7.0%)	0.292

The abbreviations are the same as Table 1.

Bold annotations indicate statistical differences.

Next, we included variables with p < 0.1 in the univariate analysis into the multivariate logistic regression model. Multivariate logistic analysis before PSM indicated that multi-site infection [OR (95%CI) = 2.759 (1.118–6.410), p = 0.018] and CRPA infection [OR (95%CI) = 3.333 (1.217–9.124), p = 0.019] were independent factors associated with the microbial cure of PMB. Multivariate logistic analysis after PSM revealed that CRPA infection [OR (95%CI) = 9.628 (1.515–80.552), p = 0.037] and number of antibiotics [OR (95%CI) = 0.279 (0.105–0.742), p = 0.011] were independent factors associated with the microbial cure of PMB (Table 4).

**TABLE 4:** Multivariate logistic analysis of PMB-associated microbial efficacy in stroke-associated pneumonia patients.

Demographics and clinical characteristics	Before PSM			After PSM		
	B	OR (95%CI)	P-value	B	OR (95%CI)	P-value
Sepsis/septic shock	0.371	1.450 (0.633–3.320)	0.380	0.232	1.261 (0.353–4.505)	0.721
Ischemic stroke	−0.366	0.693 (0.337–1.426)	0.319	−0.951	0.386 (0.107–1.395)	0.147
Multi-site infections	1.015	2.759 (1.188–6.410)	**0.018**	1.222	3.395 (0.796–14.491)	0.099
CRPA infection	1.204	3.333 (1.217–9.124)	**0.019**	2.265	9.628 (1.515–80.552)	**0.037**
CRAB infection	−0.462	0.630 (0.261–1.524)	0.305	−0.679	0.507 (0.115–2.235)	0.370
Multiple CR-GNB infections	0.260	1.297 (0.576–2.917)	0.530	0.070	1.072 (0.256–4.493)	0.924
Inhalation	−0.464	0.629 (0.290–1.364)	0.240	−0.775	0.461 (0.151–1.402)	0.172
Maintenance dose (mg)	−0.005	0.995 (0.973–1.018)	0.677	0.000	1.000 (0.965–1.037)	0.988
Treatment course (days)	0.005	1.005 (0.899–1.124)	0.926	0.011	1.011 (0.998–1.001)	0.895
Cumulative dose (mg)	0.000	1.000 (0.999–1.000)	0.417	0.000	1.000 (0.998–1.001)	0.609
Number of antibiotics	−0.365	0.694 (0.414–1.165)	0.167	−1.277	0.279 (0.105–0.742)	**0.011**

The parameters included in multivariate logistic regression were those with p < 0.1 in the univariate test. Bold font indicates data with significant differences. B indicates regression coefficient. *Data are presented as median (minimum-maximum).

### 3.5 Risk factors associated the clinical efficacy and 30-day all-cause mortality rate of PMB

Concurrently, we investigated the clinical efficacy and 30-day all-cause mortality rate of polymyxin B in the treatment of stroke-associated pneumonia. Subgroup analysis of the clinical efficacy real-world data (Supplementary Tables S1, S2) revealed that hemodynamic instability is an independent risk factor for treatment failure [OR (95%CI) = 2.979 (1.311–6.771), p = 0.009], and the combination with meropenem helps to enhance clinical outcomes [OR (95%CI) = 0.394 (0.168–0.922), p = 0.0.032]. However, after PSM, hemodynamic status and combination therapy were not statistically significant in the multivariate analysis (p > 0.05). In the subgroup analysis for 30-day all-cause mortality (Supplementary Tables S3, S4), age was identified as an independent risk factor for 30-day mortality in both real-world data and post-PSM data {[HR (95%CI) = 1.046 (1.010–1.082), p = 0.011] vs. [HR (95%CI) = 1.070 (1.014–1.129), p = 0.013]}.

## 4 Discussion

The prognosis of stroke patients is closely related to the presence of infectious complications. Among these complications, pneumonia is the most common complication in acute stroke patients, and SAP patients had a higher risk of infection with CR-GNB (Liu et al., 2018). PMB was widely used in the treatment of CR-GNB infections, but there is limited clinical data on the effectiveness of PMB in the treatment of SAP infected with CR-GNB. Herein, we evaluated the clinical and microbiological efficacy of PMB-based regimens in SAP patients with CR-GNB infection. We found that the clinical efficacy was 68.9%, and the 7-day microbial efficacy and the microbial cure rate were 25.5% and 37.8%, respectively. The 30-day all-cause mortality was 14.8% and the AKI rate was 34.7%. Additionally, we identified independent risk factors associated with efficacy.

Infections caused by CR-GNB are known to prolong hospital stays and lead to high mortality (Ye et al., 2022). Treatment options remain limited, particularly in China, with tigecycline, polymyxins, and ceftazidime/avibactam being the primary options (Qu et al., 2022). PMB is effective against all types of carbapenems-producing CR-GNBs, such as CRKP, CRPA, and CRAB (Nang et al., 2021). Previous studies have analyzed the clinical efficacy of PMB against CR-GNB infection, with reported efficacy rates ranging from 25.1% to 66.7% (Zeng et al., 2020; Qu et al., 2022; Tang et al., 2023). A study about PMB treating 105 cases of CR-GNB nosocomial pneumonia reported a clinical efficacy rate was 66.7% (Tang et al., 2023). Another study on 107 patients with nosocomial pneumonia treated with intravenous PMB also has a clinical success rate of 62.6% (Zeng et al., 2020). Our study’s clinical efficacy rate of 68.9% aligns closely with these prior findings, underscoring the potential of PMB as a therapeutic option in this challenging patient population.

In a substantial number of patients, the lung concentration of PMB is not ideal, highlighting the need for combination therapy or adjunctive atomized inhalation of PMB (Amaya-Villar and Garnacho-Montero, 2019). Inhalation of PMB is beneficial for patients who do not achieve the target concentration with intravenous administration (Tang et al., 2023). A comparative study about the treatment of pneumonia caused by drug-resistant GNBs found that both inhaled PMB and intravenous PMB showed high microbial clearance rates but with no significant difference in 28-day mortality (Shi et al., 2023). A multicenter case-control study comparing the efficacy and safety of a combined inhaled and intravenous PMB *versus* intravenous PMB alone in patients with extensively drug-resistant GNB-infected ventilator-associated pneumonia, found that combination therapy did not significantly improve the efficacy and microbial clearance compared to intravenous PMB alone (Liu et al., 2022). There are conflicting views on the role of aerosolized antibiotics as an adjunct to intravenous antibiotics. In the Infectious Diseases Society of America 2023 Guidelines for the treatment of antimicrobial resistant gram-negative infections, the panel does not recommend aerosolized antibiotics for the treatment of respiratory infections caused by CRPA or CRAB (Tamma et al., 2023), citing a lack of clear clinical benefits from aerosolized therapy in clinical trials. Concerns include the potential for uneven distribution of PMB within infected lung regions and adverse respiratory complications, such as bronchospasm (Maselli et al., 2017). Conversely, the International consensus guidelines for optimal use of polymyxins recommend that patients requiring intravenous polymyxin therapy for extensively drug-resistant GNB-infected ventilator-associated pneumonia should receive adjunctive polymyxin inhalation therapy, and either colistin or PMB is appropriate (Tsuji et al., 2019). In our study, there was no significant difference in clinical efficacy and 30-day all-cause mortality between the PMB combined inhalation group and non-combined inhalation group. However, patients treated with atomized PMB had higher microbial efficacy in the univariate analysis after adjusting the propensity score, suggesting the effectiveness of atomized PMB therapy. Therefore, appropriate inhalation of PMB therapy is important to improve microbial clearance in the lungs (Liu et al., 2022). Interestingly, when comparing the PMB combined inhalation group with the non-combined inhalation group, the incidence of AKI was higher in the combined inhalation group than in the non-combined inhalation group (before PSM: 45.8% vs. 31.1%, after PSM: 44.4% vs. 37.8%, p > 0.05). Although there was no significant difference, this may be attributed to the higher rate of loading dose use in the inhalation group in the cases we included (81.3% vs. 61.5%, p = 0.062), as well as a higher proportion of loading dose ≥ 2 mg/kg (45.8% vs. 33.1%, p = 0.475). Further research is essential to optimize dosing regimens for critically ill patients through reliable pharmacokinetics data on aerosol inhalation of PMB.

Clearing CR-GNB strains, especially in SAP patients, is challenging due to factors such as consciousness disturbance and long-term bed rest, which can lead to hypostatic pneumonia (Gittins et al., 2023). Additionally, some patients may experience reflux aspiration and exhibit poor airway clearance ability (Gittins et al., 2023). Our data showed that the microbial clearance rate of PMB was only 37.8%. Logistic regression results identified multi-site infection, CRPA infection, and number of antibiotics as independent positive factors influencing microbial clearance in PMB-based regimens. Our previous comparative study also noted that CRPA is more difficult to clear than CRKP and CRAB (Lu et al., 2021b). *In vitro* experiments showed that although CRPA was more than 90% sensitive to PMB, some CRPA strains were resistant to PMB (Chen et al., 2020). In summary, the microbial efficacy of PMB in treating CR-GNB-infected SAP patients was related to the infection sites, types of CR-GNB, details of medication, and combination of antibiotics.

This study represents the first study about the efficacy of PMB in SAP patients infected with CR-GNB. Although the data were collected at multicenter, this retrospective study is limited by the small sample size, with only 24.5% of patients in the PMB combined inhalation group. Factors associated with efficacy may require more detailed clinicopathological and physiological data. In addition, potential subjective biases in physicians’ evaluation of clinical efficacy should be considered. Moreover, the causes of AKI are very complex, and drugs may only be one aspect of the influencing factors. Although we have tried our best to judge the possibility of PMB-associated AKI, there is still a lack of a more precise definition. Further multicenter prospective cohort studies with larger sample sizes are needed to determine the efficacy of PMB in treating SAP infected with CR-GNB.

## 5 Conclusion

Although there was no significant difference in clinical efficacy between atomized and non-atomized PMB in SAP patients infected with CR-GNB infection, patients treated with atomized PMB had higher microbial efficacy. Multi-site infections, CRPA infection, and numbers of antibiotics were independent risk factors for microbial clearance in PMB treatment. PMB aerosol therapy is recommended, and personalized PMB-based regimens for individual patients may be beneficial in treating SAP patients infected with CR-GNB.

## Data Availability

The original contributions presented in the study are included in the article/Supplementary Material, further inquiries can be directed to the corresponding author.
