# Supplementary material for: The efficacy of polymyxin B in treating stroke-associated pneumonia with carbapenem-resistant Gram-negative bacteria infections: a multicenter real-world study using propensity score matching
Source: Front Pharmacol. 2025 Mar 20;16:1413563. doi: 10.3389/fphar.2025.1413563 (PMC11965127; doi:10.3389/fphar.2025.1413563)
Supplement: Supplementary file 1 [file DataSheet1.docx]

**
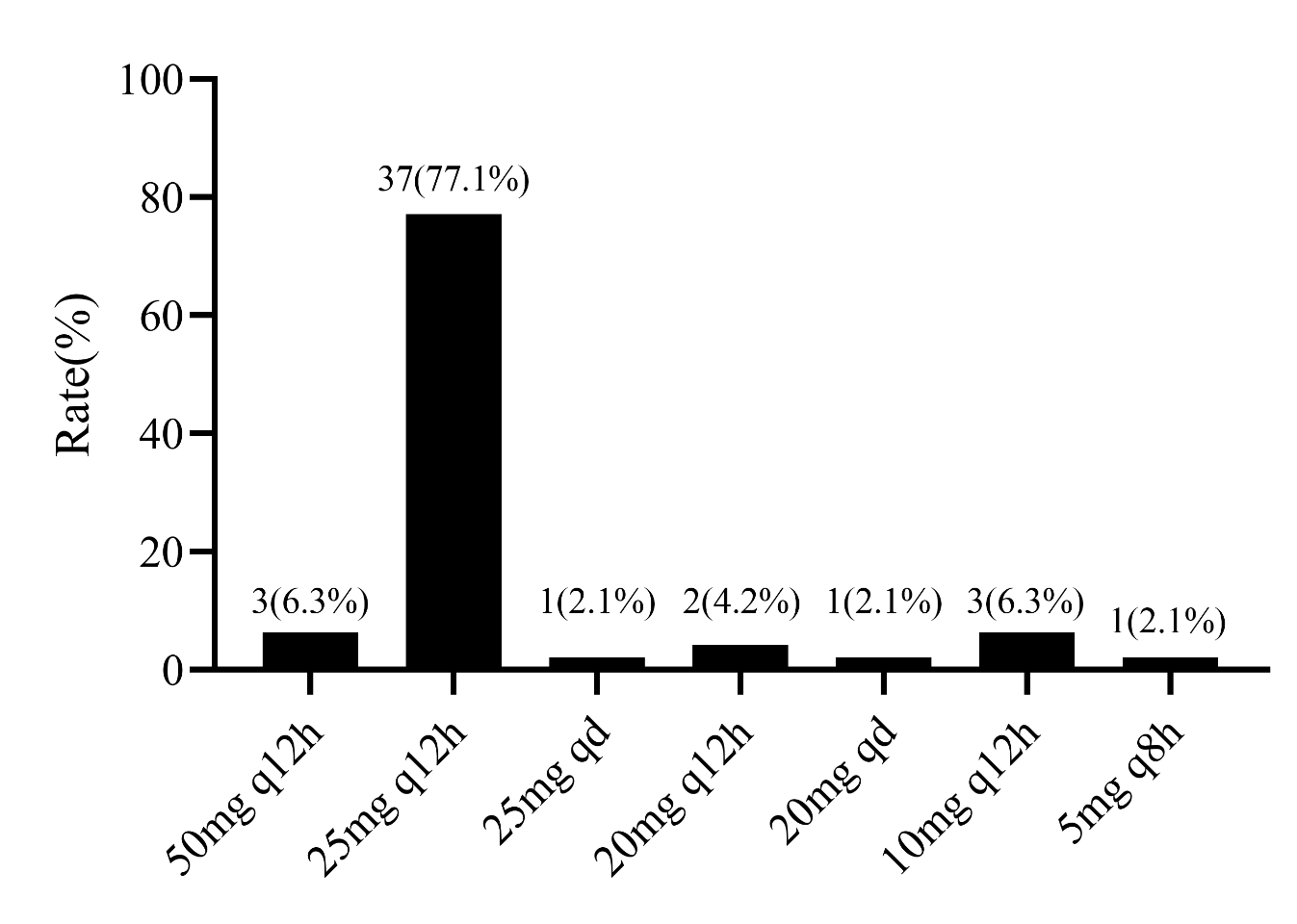
**

**Figure S1** The details of Polymyxin B inhalation administration

**Table S1** Univariate analysis of clinical efficacy of PMB in the treatment of stroke-associated pneumonia with CR-GNB infection

| **Demographics and clinical characteristics** | **Before PSM** | | | **After PSM** | | |
| --- | --- | --- | --- | --- | --- | --- |
|  | **Effective  (N=135)** | **Ineffective  (N=61)** | **P-value** | **Effective  (N=65)** | **Ineffective  (N=25)** | **P-value** |
| Age (years) | 61.0(49.0-73.0) | 68.0(55.0-79.5) | **0.015** | 68.0(52.0-76.0) | 72.0(55.5-79.5) | 0.404 |
| Gender (male) | 105(77.8%) | 50(82.0%) | 0.504 | 49(75.4%) | 22(88.0%) | 0.305 |
| Mechanical ventilation | 91(67.4%) | 49(80.3%) | **0.064** | 55(84.6%) | 24(96.0%) | 0.264 |
| Vasoactive drugs | 49(36.3%) | 39(63.9%) | **<0.001** | 28(43.1%) | 18(72.0%) | **0.014** |
| ICU administration | 114(84.4%) | 46(75.4%) | 0.130 | 61(93.8%) | 23(92.0%) | 0.668 |
| Hospital stays (days) | 39.0(23.0-55.0) | 30.0(17.0-57.5) | **0.093** | 39.0(23.0-58.5) | 27.0(16.0-49.5) | 0.182 |
| APACHE II score | 19.6±5.6 | 19.6±6.5 | 0.634 | 20.2±6.1 | 18.5±6.2 | 0.231 |
| Creatinine before treatment (µmol/L) | 72.1(54.5-109.0) | 71.5(48.1-132.5) | 0.822 | 80.1(54.8-117.2) | 91.0(57.4-174.2) | 0.167 |
| Sepsis/septic shock | 31(23.0%) | 22(36.1%) | **0.056** | 21(32.3%) | 14(56.0%) | **0.039** |
| **Comorbidity** |  |  |  |  |  |  |
| Stroke type |  |  | **0.047** |  |  | 0.276 |
| Ischemic | 59(43.7%) | 36(59.0%) |  | 29(44.6%) | 8(32.0%) |  |
| Hemorrhagic | 76(56.3%) | 25(41.0%) |  | 36(55.4%) | 17(68.0%) |  |
| Hypoproteinemia | 45(33.0%) | 11(18.0%) | **0.028** | 24(36.9%) | 5(20.0%) | 0.124 |
| Urinary system disease | 24(17.8%) | 5(8.2%) | **0.080** | 16(24.6%) | 0(0.0%) | **0.015** |
| Diabetes mellitus | 37(27.4%) | 13(21.3%) | 0.365 | 20(30.8%) | 4(16.0%) | 0.156 |
| Digestive system diseases | 50(37.0%) | 12(19.7%) | **0.016** | 27(41.5%) | 5(20.0%) | 0.056 |
| Cardiovascular diseases | 95(70.4%) | 38(62.3%) | 0.262 | 46(70.8%) | 14(56.0%) | 0.183 |
| Malignancy | 17(12.6%) | 8(13.1%) | 0.919 | 5(7.7%) | 4(16.0%) | 0.433 |
| **Infection sits** |  |  |  |  |  |  |
| Multi-site infections | 37(27.4%) | 12(19.7%) | 0.247 | 19(29.2%) | 4(16.0%) | 0.308 |
| Blood | 13(9.6%) | 5(8.2%) | 0.748 | 10(15.4%) | 1(4.0%) | 0.264 |
| Abdominal | 1(0.7%) | 3(4.9%) | **0.090** | 1(1.5%) | 1(4.0%) | 0.481 |
| Urinary tract | 13(9.6%) | 4(6.6%) | 0.665 | 8(12.3%) | 2(8.0%) | 0.835 |
| Central nervous system | 15(11.1%) | 4(6.6%) | 0.461 | 6(9.2%) | 0(0.0%) | 0.271 |
| Sin and soft tissue | 4(3.0%) | 0(0.0%) | 0.312 | 0 | 0 | - |
| **Pathogens** |  |  |  |  |  |  |
| CRKP | 34(25.2%) | 20(32.8%) | 0.270 | 22(33.8%) | 9(36.0%) | 0.847 |
| CRPA | 104(77.0%) | 38(62.3%) | **0.032** | 14(21.5%) | 10(40.0%) | **0.076** |
| CRAB | 5(3.7%) | 3(4.9%) | 0.691 | 52(80.0%) | 14(56.0%) | **0.021** |
| Other CREs | 49(36.3%) | 21(34.4%) | 0.800 | 4(6.2%) | 2(8.0%) | 0.688 |
| Multiple CR-GNB infections | 48(35.6%) | 24(39.3%) | 0.610 | 23(35.4%) | 9(36.0%) | 0.956 |
| **PMB Treatment** |  |  |  |  |  |  |
| Inhalation (%) | 36(26.7%) | 12(19.7%) | 0.292 | 35(53.8%) | 10(40.0%) | 0.239 |
| Inhalation therapy regimen(days) | 0.0(0.0-3.0) | 0.0(0.0-0.0) | 0.234 | 3.0(0.0-8.0) | 0.0(0.0-5.5) | 0.120 |
| Loading dose (%) | 97(71.9%) | 33(54.1%) | **0.015** | 55(84.6%) | 19(76.0%) | 0.338 |
| The loading dose (mg) | 100.0(100.0-130.0) | 100.0(50.0-100.0) | **0.005** | 100.0(100.0-150.0) | 100.0(87.5-150.0) | 0.823 |
| Maintenance dose (mg) | 75.0(50.0-80.0) | 50.0(50.0-75.0) | 0.143 | 75.0(50.0-75.0) | 75.0(50.0-75.0) | 0.951 |
| The loading dose≥2mg/kg | 55(40.7%) | 16(26.2%) | **0.050** | 27(41.5%) | 10(40.0%) | 0.894 |
| Maintenance dose≥1.25mg/kg | 63(46.7%) | 24(39.3%) | 0.339 | 29(44.6%) | 13(52.0%) | 0.529 |
| Treatment course (days) | 11.0(7.0-15.0) | 8.0(5.0-13.8) | 0.234 | 11.0(7.0-15.8) | 7.0(4.0-13.0) | **0.013** |
| Cumulative dose (mg) | 1425.0(945.0-2275.0) | 1075.0(737.5-1687.5) | **0.017** | 1425.0(875.0-2362.5) | 1050.0(662.5-1350.0) | **0.023** |
| Number of antibiotics | 1.0(1.0-2.0)) | 1.0(1.0-1.0) | **0.028** | 1.0(1.0-2.0) | 1.0(1.0-1.5) | **0.072** |
| β-lactam | 64(47.4%) | 18(29.5%) | **0.019** | 31(47.7%) | 8(32.0%) | 0.178 |
| Tigecycline | 25(18.5%) | 18(29.5%) | **0.085** | 21(32.3%) | 10(40.0%) | 0.492 |
| Carbapenem | 70(51.9%) | 22(36.1%) | **0.040** | 34(52.3%) | 9(36.0%) | 0.165 |
| Only PMB | 23(17.0%) | 14(23.0%) | 0.327 | 2(3.1%) | 2(8.0%) | 0.310 |

The abbreviations are the same as Table 1.

**Table S2** Multivariate logistic analysis of PMB-associated clinical efficacy in stroke-associated pneumonia patients

| **Demographics and clinical characteristics** | **Before PSM** | | | **After PSM** | | |
| --- | --- | --- | --- | --- | --- | --- |
|  | **B** | **OR (95%CI)** | **P-value** | **B** | **OR (95%CI)** | **P-value** |
| Age (years) | 0.023 | 1.023(0.997-1.050) | 0.086 | 0.006 | 1.006(0.963-1.050) | 0.796 |
| Vasoactive drugs | 1.092 | 2.979(1.311-6.771) | **0.009** | 1.217 | 3.378(0.913-12.494) | 0.068 |
| Hemorrhagic | 0.201 | 1.223(0.542-2.759) | 0.628 | 0.333 | 1.396(0.353-5.513) | 0.634 |
| Hypoproteinemia | -0.426 | 0.653(0.278-1.535) | 0.328 | -0.294 | 0.745(0.196-2.838) | 0.666 |
| Digestive system diseases | -0.571 | 0.565(0.249-1.282) | 0.172 | -1.025 | 0.359(0.089-1.441) | 0.148 |
| CRPA | 0.602 | 1.826(0.827-4.034) | 0.136 | 1.441 | 4.225(1.086-16.437) | **0.038** |
| Loading dose (%) | -0.692 | 0.501(0.120-2.086) | 0.342 | -1.277 | 0.279(0.031-2.526) | 0.256 |
| The loading dose (mg) | 0.001 | 1.001(0.975-1.028) | 0.924 | 0.008 | 1.008(0.970-1.047) | 0.696 |
| Maintenance dose (mg) | 0.002 | 1.002(0.973-1.032) | 0.896 | 0.008 | 1.008(0.965-1.053) | 0.724 |
| The loading dose≥2mg/kg | -0.718 | 0.488(0.173-1.372) | 0.174 | -0.389 | 0.678(0.131-3.500) | 0.642 |
| Cumulative dose (mg) | 0.000 | 1.000(0.999-1.000) | 0.378 | 0.000 | 1.000(0.999-1.000) | 0.356 |
| Number of antibiotics | -0.100 | 0.905(0.459-1.784) | 0.773 | -0.447 | 0.639(0.211-1.942) | 0.430 |
| β-lactam | -1.245 | 0.288(0.114-0.726) | **0.008** | -0.814 | 0.443(0.099-1.976) | 0.286 |
| Carbapenem | -0.932 | 0.394(0.168-0.922) | **0.032** | -0.620 | 0.538(0.132-2.186) | 0.386 |

The parameters included in multivariate logistic regression were those with p < 0.050 in the univariate test. Bold font indicates data with significant differences. B indicates regression coefficient. *Data are presented as median (minimum-maximum).

**Table S3** Univariate analysis of 30-day all-cause mortality rate of PMB in the treatment of stroke-associated pneumonia with CR-GNB infection

| **Demographics and clinical characteristics** | **Before PSM** | | | **After PSM** | | |
| --- | --- | --- | --- | --- | --- | --- |
|  | **Effective  (N=167)** | **Ineffective  (N=29)** | **P-value** | **Effective  (N=72)** | **Ineffective  (N=18)** | **P-value** |
| Age (years) | 61.0(50.0-73.0) | 75.0(59.0-81.0) | **0.001** | 66.0(52.0-75.0) | 76.0(71.0-81.0) | 0.004 |
| Gender (male) | 134(80.2%) | 21(72.4%) | 0.339 | 59(81.9%) | 12(66.7%) | 0.155 |
| Mechanical ventilation | 114(68.3%) | 26(89.7%) | **0.033** | 63(87.5%) | 16(88.9%) | 1.000 |
| Vasoactive drugs | 70(41.9%) | 18(62.1%) | **0.044** | 33(45.8%) | 13(72.2%) | **0.045** |
| ICU administration | 136(81.4%) | 24(82.8%) | 0.865 | 66(91.7%) | 18(100.0%) | 0.460 |
| Hospital stays (days) | 38.0(22.0-58.0) | 27.0(16.0-37.0) | **0.005** | 40.5(23.0-61.8) | 22.0(12.8-36.8) | 0.003 |
| APACHE II score | 19.2±5.5 | 21.8±7.4 | 0.137 | 19.3±5.5 | 21.8±8.0 | 0.271 |
| Creatinine before treatment (µmol/L) | 71.5(53.9-108.5) | 80.3(47.3-152.9) | 0.716 | 79.7(54.8-112.2) | 114.0(55.8-185.0) | 0.180 |
| Sepsis/septic shock | 38(22.8%) | 15(51.7%) | **0.001** | 22(30.6%) | 13(72.2%) | **0.001** |
| **Comorbidity** |  |  |  |  |  |  |
| Stroke type |  |  | **0.005** |  |  | **0.004** |
| Ischemic | 74(44.3%) | 21(72.4%) |  | 37(51.4%) | 16(88.9%) |  |
| Hemorrhagic | 93(55.7%) | 8(27.6%) |  | 35(48.6%) | 2(11.1%) |  |
| Hypoproteinemia | 52(31.1%) | 4(13.8%) | 0.092 | 26(36.1%) | 3(16.7%) | 0.195 |
| Urinary system disease | 25(15.0%) | 4(13.8%) | 1.000 | 16(22.2%) | 0(0.0%) | **0.063** |
| Diabetes mellitus | 42(251%) | 8(27.6%) | 0.781 | 21(29.2%) | 3(16.7%) | 0.439 |
| Digestive system diseases | 514(32.3%) | 8(27.6%) | 0.612 | 28(38.9%) | 4(22.2%) | 0.296 |
| Cardiovascular diseases | 110(65.9%) | 23(79.3%) | 0.153 | 46(63.9%) | 14(77.8%) | 0.402 |
| Malignancy | 21(12.6%) | 4(13.8%) | 0.856 | 8(11.1%) | 1(5.6%) | 0.792 |
| **Infection sits** |  |  |  |  |  |  |
| Multi-site infections | 43(25.7%) | 6(20.7%) | 0.561 | 19(26.4%) | 4(22.2%) | 0.952 |
| Blood | 15(9.0%) | 3(10.3%) | 0.815 | 8(11.1%) | 3(16.7%) | 0.809 |
| Abdominal | 3(2.8%) | 1(3.4%) | 1.000 | 1(1.4%) | 1(5.6%) | 0.362 |
| Urinary tract | 16(9.6%) | 1(3.4%) | 0.468 | 10(13.9%) | 0(0.0%) | 0.208 |
| Central nervous system | 18(10.8%) | 1(3.4%) | 0.218 | 6(8.3%) | 0(0.0%) | 0.460 |
| Sin and soft tissue | 4(2.4%) | 0(0.0%) | 1.000 | 0 | 0 | - |
| **Pathogens** |  |  |  |  |  |  |
| CRKP | 60(35.9%) | 12(41.4%) | 0.574 | 24(33.3%) | 7(38.9%) | 0.657 |
| CRPA | 51(30.5%) | 3(10.3%) | **0.043** | 22(30.6%) | 2(11.1%) | 0.170 |
| CRAB | 122(73.1%) | 20(69.0%) | 0.649 | 55(76.4%) | 11(61.1%) | 0.190 |
| Other CREs | 6(3.6%) | 2(6.9%) | 0.748 | 4(5.6%) | 2(11.1%) | 0.751 |
| Multiple CR-GNB infections | 62(37.1%) | 8(27.6%) | 0.322 | 28(38.9%) | 4(22.2%) | 0.296 |
| **PMB Treatment** |  |  |  |  |  |  |
| Inhalation (%) | 40(83.3%) | 8(27.6%) | 0.674 | 37(51.4%) | 8(44.4%) | 0.598 |
| Inhalation therapy regimen(days) | 0.0(0.0-0.0) | 0.0(0.0-2.5) | 0.914 | 2.0(0.0-8.0) | 0.0(0.0-5.0) | 0.273 |
| Loading dose (%) | 105(62.9%) | 25(86.2%) | **0.025** | 58(80.6%) | 16(21.6%) | 0.629 |
| The loading dose (mg) | 100.0(100.0-125.0) | 100.0(100.0-150.0) | 0.415 | 100.0(100.0-150.0) | 100.0(93.8-127.5) | 0.369 |
| Maintenance dose (mg) | 75.0(50.0-90.0) | 50.0(50.0-75.0) | 0.311 | 75.0(50.0-75.0) | 50.0(50.0-75.0) | 0.072 |
| The loading dose≥2mg/kg | 61(36.5%) | 10(34.5%) | 0.833 | 31(43.1%) | 6(33.3%) | 0.453 |
| Maintenance dose≥1.25mg/kg | 77(46.1%) | 10(34.5%) | 0.245 | 37(51.4%) | 5(27.8%) | 0.073 |
| Treatment course (days) | 11.0(7.0-15.0) | 7.0(6.0-10.5) | **0.003** | 11.8(7.0-16.0) | 7.0(5.8-10.3) | **0.007** |
| Cumulative dose (mg) | 1400.0(850.0-2300.0) | 1050.0(750.0-1262.5) | **0.002** | 1437.5(850.0-2443.8) | 962.5(625.0-1093.8) | **0.002** |
| Number of antibiotics | 1.0(1.0-2.0) | 1.0(1.0-1.0) | 0.282 | 1.0(1.0-2.0) | 1.0(1.0-1.0) | **0.027** |
| β-lactam | 71(42.5%) | 11(37.9%) | 0.644 | 35(48.6%) | 4(22.2%) | 0.079 |
| Tigecycline | 33(19.8%) | 10(34.5%) | 0.077 | 22(30.6%) | 9(50.0%) | 0.120 |
| Carbapenem | 81(48.5%) | 11(37.9%) | 0.292 | 36(50.0%) | 7(38.9%) | 0.399 |
| Only PMB | 30(18.0%) | 7(24.1%) | 0.433 | 3(4.2%) | 1(5.6%) | 1.000 |

The abbreviations are the same as Table 1.

**Table S4** COX analysis of PMB-associated 30-day all-cause mortality rate in stroke-associated pneumonia patients

| **Demographics and clinical characteristics** | **Before PSM** | | | **After PSM** | | |
| --- | --- | --- | --- | --- | --- | --- |
|  | **B** | **HR (95%CI)** | **P-value** | **B** | **HR (95%CI)** | **P-value** |
| Sepsis/septic shock | 0.710 | 2.033(0.852-4.851) | 0.110 | 1.327 | 3.771(0.846-16.806) | 0.082 |
| Age (years) | 0.045 | 1.046(1.010-1.082) | **0.011** | 0.068 | 1.070(1.014-1.129) | **0.013** |
| Mechanical ventilation | 1.008 | 2.739(0.707-10.608) | 0.145 | -0.454 | 0.635(0.082-4.940) | 0.665 |
| Vasoactive drugs | -0.125 | 0.882(0.357-2.181) | 0.786 | 0.233 | 1.262(0.320-4.974) | 0.739 |
| Hospital stays (days) | -0.009 | 0.991(0.977-1.005) | 0.187 | -0.015 | 0.985(0.967-1.003) | 0.111 |
| Hemorrhagic | -0.170 | 0.843(0.327-2.176) | 0.725 | -0.175 | 0.839(0.135-5.219) | 0.851 |
| CRPA | -1.053 | 0.349(0.098-1.244) | 0.104 | -0.816 | 0.442(0.092-2.128) | 0.309 |
| Loading dose (%) | 1.697 | 5.456(1.829-16.271) | **0.002** | 1.044 | 2.839(0.610-13.219) | 0.184 |
| Treatment course (days) | -0.088 | 0.916(0.785-1.069) | 0.264 | 0.021 | 1.021(0.839-1.242) | 0.835 |
| Cumulative dose (mg) | 0.000 | 1.000(0.999-1.000) | 0.348 | -0.001 | 0.999(0.997-1.000) | 0.112 |

The parameters included in multivariate logistic regression were those with p < 0.050 in the univariate test. Bold font indicates data with significant differences. B indicates regression coefficient. *Data are presented as median (minimum-maximum).
